# Bis(acetyl­acetonato-κ^2^ *O*,*O*′)(methanol-κ*O*)(thio­cyanato-κ*N*)manganese(III)

**DOI:** 10.1107/S1600536808031589

**Published:** 2008-10-04

**Authors:** Shuang-Ming Meng, Hai Xie, Yue-Qin Fan, Yong Guo

**Affiliations:** a College of Chemistry and Chemical Engineering, Shanxi Datong University, Shanxi 037009, People’s Republic of China

## Abstract

In the title complex, [Mn(C_5_H_7_O_2_)_2_(NCS)(CH_4_O)], the Mn^III^ atom has a slightly distorted octa­hedral coordination formed by five O atoms and one N atom. The equatorial positions are occupied by four O atoms of two acetyl­acetonate ligands, while the axial positions are occupied by the N atom of the thio­cyanate anion and the O atom of the methanol mol­ecule. In the crystal structure, complex mol­ecules are linked by an inter­molecular O—H⋯S hydrogen bond, forming a chain running along [101].

## Related literature

For the synthesis, see: Stults *et al.* (1975 ▶). For related structures, see: Stults *et al.* (1979 ▶); Swarnabala *et al.* (1994 ▶).
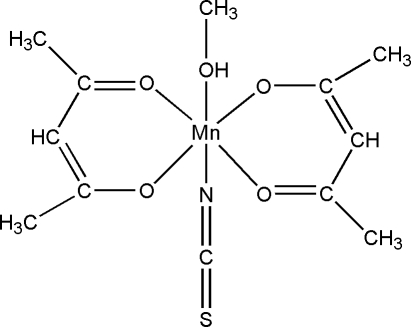


## Experimental

### 

#### Crystal data

[Mn(C_5_H_7_O_2_)_2_(NCS)(CH_4_O)]
*M* *_r_* = 343.27
Monoclinic, 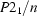

*a* = 7.4795 (13) Å
*b* = 12.420 (2) Å
*c* = 17.586 (3) Å
β = 98.673 (4)°
*V* = 1614.9 (5) Å^3^
*Z* = 4
Mo *K*α radiation
μ = 0.96 mm^−1^
*T* = 293 (2) K
0.21 × 0.19 × 0.15 mm

#### Data collection

Bruker APEXII CCD area-detector diffractometer
Absorption correction: multi-scan (**SADABS**; Bruker, 2000 ▶) *T*_min_ = 0.824, *T*_max_ = 0.869
7925 measured reflections
2830 independent reflections
1276 reflections with *I* > 2σ(*I*)
*R*_int_ = 0.112

#### Refinement

*R*[*F* ^2^ > 2σ(*F* ^2^)] = 0.058
*wR*(*F* ^2^) = 0.148
*S* = 0.91
2830 reflections
189 parameters
H atoms treated by a mixture of independent and constrained refinement
Δρ_max_ = 0.33 e Å^−3^
Δρ_min_ = −0.33 e Å^−3^

### 

Data collection: *APEX2* (Bruker, 2004 ▶); cell refinement: *APEX2*; data reduction: *SAINT-Plus* (Bruker, 2004 ▶); program(s) used to solve structure: *SHELXS97* (Sheldrick, 2008 ▶); program(s) used to refine structure: *SHELXL97* (Sheldrick, 2008 ▶); molecular graphics: *SHELXTL* (Sheldrick, 2008 ▶); software used to prepare material for publication: *SHELXTL*.

## Supplementary Material

Crystal structure: contains datablocks I, global. DOI: 10.1107/S1600536808031589/is2333sup1.cif

Structure factors: contains datablocks I. DOI: 10.1107/S1600536808031589/is2333Isup2.hkl

Additional supplementary materials:  crystallographic information; 3D view; checkCIF report

## Figures and Tables

**Table 1:** Hydrogen-bond geometry (Å, °)

*D*—H⋯*A*	*D*—H	H⋯*A*	*D*⋯*A*	*D*—H⋯*A*
O5—H5*A*⋯S1^i^	0.78 (7)	2.51 (6)	3.281 (4)	168 (7)

Symmetry code: (i) 
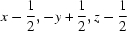
.

**Fig. 1.**
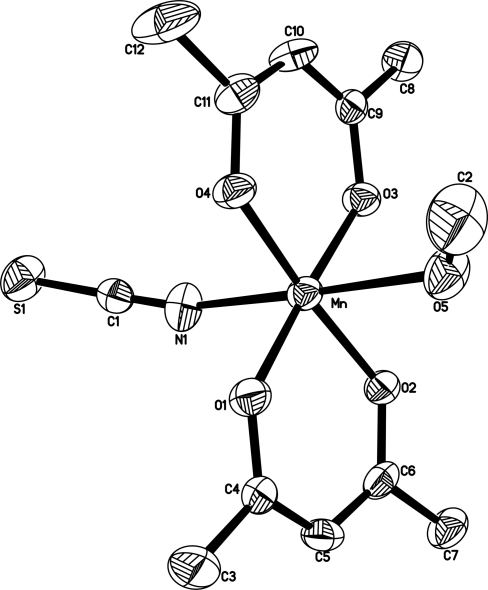
A view of the title complex with the atom-labelling scheme. Displacement ellipsoids are drawn at the 30% probability level. H atoms have been omitted.

## supplementary crystallographic information

### Comment

Octahedral complexes of high-spin Mn^III^ are good examples for investigating the Jahn-Teller distortions, because their geometry are always distorted from the ideal octahedron to the distorted one by the axial ligands. Here, we report the structure of an octahedral Mn^III^ complex, whose synthesis has been reported early (Stults *et al.*, 1975).

The molecular structure of the title complex is shown in Figure 1. The Mn^III^ atom is six coordinated by five O atoms and one N atom. The geometry can be described as a distorted octahedron. Four equational positions are occupied by four O atoms coming from two acetylacetonate ligands with the average Mn—O bond length 1.909 Å, which is in agreement well with the corresponding distance in [Mn(acac)_2_(OH_2_)_2_]ClO_4_].2H_2_O. (Swarnabala *et al.*, 1994). One SCN^-^ ion and one methanol molecule are coordinated to the Mn^III^ atom with *trans* positions, so that forming an octahedral geometry. The distance of Mn—O_methanol_ [2.289 (5) Å] is obviously longer than the bond lengths of Mn—O_acetylacetonate_. The bond length of Mn—N_SCN_ is 2.187 (6) Å, which is also consistent with that found in [Mn(acac)_2_(SCN)] (Stults *et al.*, 1979). In the crystal structure, a molecular chain along the [101] direction is formed by an intermolecular H-bond between the O atom of the methanol molecule and the S atom of the SCN^-^ ion (Table 1).

### Experimental

The title complex was synthesized according to the literature method (Stults *et al.* 1975). The single crystals suitable for X-ray diffraction were grown from a methanol solution after the solvent was partial evaporated. Anal. Calcd for C_12_H_18_MnNO_5_S: C 41.99, H 5.29, N 4.08; found: C 42.04, H 5.26, N, 4.11.

### Refinement

The O-bound H atom of the methanol molecule was located in a difference Fourier map and its coordinates were refined, with *U*_iso_(H) = 1.5*U*_eq_(O). The H atoms bound to C atoms were placed geometrically (C—H = 0.93–0.96 Å) and were refined as riding, with *U*_iso_(H) = 1.2*U*_eq_(C) or 1.5*U*_eq_(methyl C).

### Crystal data

**Table:**

[Mn(C_5_H_7_O_2_)_2_(NCS)(CH_4_O)]	*F*(000) = 712
*M**_r_* = 343.27	*D*_x_ = 1.412 Mg m^−^^3^
Monoclinic, *P*2_1_/*n*	Mo *K*α radiation, λ = 0.71073 Å
Hall symbol: -P2yn	Cell parameters from 1347 reflections
*a* = 7.4795 (13) Å	θ = 2.8?–16.2°
*b* = 12.420 (2) Å	µ = 0.96 mm^−^^1^
*c* = 17.586 (3) Å	*T* = 293 K
β = 98.673 (4)°	Block, brown
*V* = 1614.9 (5) Å^3^	0.21 × 0.19 × 0.15 mm
*Z* = 4	

### Data collection

**Table:**

Bruker APEXII CCD area-detector diffractometer	2830 independent reflections
Radiation source: fine-focus sealed tube	1276 reflections with *I* > 2σ(*I*)
graphite	*R*_int_ = 0.112
φ and ω scans	θ_max_ = 25.0°, θ_min_ = 2.0°
Absorption correction: multi-scan (*SADABS*; Bruker, 2000)	*h* = −8→8
*T*_min_ = 0.824, *T*_max_ = 0.869	*k* = −14→14
7925 measured reflections	*l* = −16→20

### Refinement

**Table:**

Refinement on *F*^2^	Primary atom site location: structure-invariant direct methods
Least-squares matrix: full	Secondary atom site location: difference Fourier map
*R*[*F*^2^ > 2σ(*F*^2^)] = 0.058	H atoms treated by a mixture of independent and constrained refinement
*wR*(*F*^2^) = 0.148	*w* = 1/[σ^2^(*F*_o_^2^) + (0.0541*P*)^2^] where *P* = (*F*_o_^2^ + 2*F*_c_^2^)/3
*S* = 0.91	(Δ/σ)_max_ < 0.001
2830 reflections	Δρ_max_ = 0.33 e Å^−^^3^
189 parameters	Δρ_min_ = −0.33 e Å^−^^3^
0 restraints	

### Special details

**Table:**

Geometry. All esds (except the esd in the dihedral angle between two l.s. planes) are estimated using the full covariance matrix. The cell esds are taken into account individually in the estimation of esds in distances, angles and torsion angles; correlations between esds in cell parameters are only used when they are defined by crystal symmetry. An approximate (isotropic) treatment of cell esds is used for estimating esds involving l.s. planes.
Refinement. Refinement of F^2^ against ALL reflections. The weighted R-factor wR and goodness of fit S are based on F^2^, conventional R-factors R are based on F, with F set to zero for negative F^2^. The threshold expression of F^2^ > 2sigma(F^2^) is used only for calculating R-factors(gt) etc. and is not relevant to the choice of reflections for refinement. R-factors based on F^2^ are statistically about twice as large as those based on F, and R- factors based on ALL data will be even larger.

### Fractional atomic coordinates and isotropic or equivalent isotropic displacement parameters (Å^2^)

**Table:**

	*x*	*y*	*z*	*U*_iso_*/*U*_eq_	
Mn	0.86466 (12)	0.33696 (7)	0.64488 (5)	0.0398 (3)	
S1	1.1860 (3)	0.34036 (15)	0.90976 (10)	0.0749 (7)	
O1	1.0806 (5)	0.3054 (3)	0.6035 (2)	0.0504 (12)	
O2	0.7813 (5)	0.1923 (3)	0.6275 (2)	0.0446 (11)	
O3	0.6418 (5)	0.3674 (3)	0.6806 (2)	0.0458 (11)	
O4	0.9347 (5)	0.4844 (3)	0.6515 (2)	0.0485 (12)	
O5	0.7201 (7)	0.3709 (4)	0.5230 (3)	0.0682 (16)	
H5A	0.701 (11)	0.325 (6)	0.491 (4)	0.102*	
C1	1.0772 (9)	0.3201 (5)	0.8239 (4)	0.0455 (17)	
C2	0.7403 (12)	0.4605 (6)	0.4764 (4)	0.100 (3)	
H2A	0.7848	0.5205	0.5082	0.150*	
H2B	0.6253	0.4788	0.4471	0.150*	
H2C	0.8245	0.4437	0.4420	0.150*	
C3	1.3234 (9)	0.2126 (6)	0.5642 (5)	0.084 (3)	
H3A	1.3176	0.2455	0.5144	0.126*	
H3B	1.3652	0.1398	0.5620	0.126*	
H3C	1.4055	0.2524	0.6011	0.126*	
C4	1.1395 (9)	0.2130 (6)	0.5877 (4)	0.0491 (17)	
C5	1.0421 (9)	0.1195 (5)	0.5885 (4)	0.0537 (19)	
H5	1.0977	0.0559	0.5768	0.064*	
C6	0.8682 (10)	0.1122 (5)	0.6054 (3)	0.0472 (18)	
C7	0.7645 (9)	0.0081 (4)	0.5966 (4)	0.061 (2)	
H7A	0.7050	−0.0029	0.6407	0.091*	
H7B	0.8463	−0.0504	0.5924	0.091*	
H7C	0.6758	0.0110	0.5511	0.091*	
C8	0.4019 (8)	0.4572 (5)	0.7255 (4)	0.058 (2)	
H8A	0.3125	0.4324	0.6842	0.088*	
H8B	0.3704	0.5280	0.7408	0.088*	
H8C	0.4067	0.4089	0.7684	0.088*	
C9	0.5827 (8)	0.4605 (5)	0.6990 (3)	0.0415 (16)	
C10	0.6804 (9)	0.5539 (5)	0.6967 (4)	0.0531 (19)	
H10	0.6281	0.6168	0.7118	0.064*	
C11	0.8462 (10)	0.5633 (5)	0.6743 (4)	0.0510 (18)	
C12	0.9380 (10)	0.6708 (5)	0.6760 (5)	0.092 (3)	
H12A	1.0490	0.6684	0.7114	0.138*	
H12B	0.8603	0.7250	0.6922	0.138*	
H12C	0.9636	0.6880	0.6255	0.138*	
N1	0.9996 (8)	0.3080 (5)	0.7624 (3)	0.0617 (17)	

### Atomic displacement parameters (Å^2^)

**Table:**

	*U*^11^	*U*^22^	*U*^33^	*U*^12^	*U*^13^	*U*^23^
Mn	0.0393 (6)	0.0339 (5)	0.0471 (6)	−0.0035 (5)	0.0093 (4)	−0.0032 (5)
S1	0.1019 (17)	0.0683 (13)	0.0490 (12)	−0.0013 (13)	−0.0060 (11)	0.0010 (11)
O1	0.043 (3)	0.046 (3)	0.065 (3)	−0.007 (2)	0.017 (2)	−0.012 (2)
O2	0.043 (3)	0.035 (2)	0.055 (3)	−0.003 (2)	0.005 (2)	−0.004 (2)
O3	0.041 (3)	0.041 (2)	0.057 (3)	−0.001 (2)	0.013 (2)	−0.003 (2)
O4	0.052 (3)	0.034 (2)	0.063 (3)	−0.003 (2)	0.016 (2)	−0.005 (2)
O5	0.090 (4)	0.069 (4)	0.043 (3)	−0.006 (3)	0.001 (3)	0.000 (3)
C1	0.041 (4)	0.042 (4)	0.056 (5)	−0.002 (3)	0.015 (4)	0.006 (4)
C2	0.135 (8)	0.097 (7)	0.061 (6)	−0.012 (6)	−0.003 (5)	0.024 (5)
C3	0.052 (5)	0.096 (6)	0.108 (7)	0.002 (4)	0.029 (5)	−0.041 (5)
C4	0.045 (5)	0.057 (4)	0.044 (4)	0.005 (4)	0.001 (3)	−0.013 (4)
C5	0.049 (5)	0.040 (4)	0.073 (5)	0.008 (4)	0.011 (4)	−0.010 (4)
C6	0.064 (5)	0.042 (4)	0.034 (4)	−0.002 (4)	0.001 (4)	−0.007 (3)
C7	0.076 (5)	0.038 (4)	0.066 (5)	−0.009 (4)	0.004 (4)	−0.007 (4)
C8	0.048 (5)	0.072 (5)	0.059 (5)	0.001 (4)	0.022 (4)	−0.014 (4)
C9	0.043 (4)	0.048 (4)	0.031 (4)	0.005 (3)	0.001 (3)	−0.006 (3)
C10	0.073 (6)	0.036 (4)	0.055 (5)	0.001 (4)	0.026 (4)	−0.011 (3)
C11	0.063 (5)	0.042 (4)	0.051 (5)	−0.010 (4)	0.019 (4)	−0.001 (4)
C12	0.109 (7)	0.039 (4)	0.140 (8)	−0.012 (5)	0.061 (6)	−0.015 (5)
N1	0.062 (4)	0.067 (4)	0.054 (4)	−0.015 (3)	0.001 (3)	0.003 (3)

### Geometric parameters (Å, °)

**Table:**

Mn—O4	1.903 (4)	C3—H3C	0.9600
Mn—O2	1.911 (4)	C4—C5	1.372 (8)
Mn—O3	1.906 (4)	C5—C6	1.380 (8)
Mn—O1	1.910 (4)	C5—H5	0.9300
Mn—N1	2.189 (6)	C6—C7	1.505 (8)
Mn—O5	2.289 (5)	C7—H7A	0.9600
S1—C1	1.623 (8)	C7—H7B	0.9600
O1—C4	1.275 (6)	C7—H7C	0.9600
O2—C6	1.280 (7)	C8—C9	1.495 (8)
O3—C9	1.296 (6)	C8—H8A	0.9600
O4—C11	1.281 (7)	C8—H8B	0.9600
O5—C2	1.405 (8)	C8—H8C	0.9600
O5—H5A	0.80 (7)	C9—C10	1.375 (8)
C1—N1	1.157 (7)	C10—C11	1.361 (8)
C2—H2A	0.9600	C10—H10	0.9300
C2—H2B	0.9600	C11—C12	1.501 (8)
C2—H2C	0.9600	C12—H12A	0.9600
C3—C4	1.494 (8)	C12—H12B	0.9600
C3—H3A	0.9600	C12—H12C	0.9600
C3—H3B	0.9600		
			
O4—Mn—O2	173.85 (19)	O1—C4—C5	123.9 (6)
O4—Mn—O3	92.02 (17)	O1—C4—C3	115.2 (6)
O2—Mn—O3	87.66 (16)	C5—C4—C3	120.8 (6)
O4—Mn—O1	88.83 (17)	C4—C5—C6	125.2 (6)
O2—Mn—O1	91.17 (17)	C4—C5—H5	117.4
O3—Mn—O1	176.83 (18)	C6—C5—H5	117.4
O4—Mn—N1	91.0 (2)	O2—C6—C5	123.6 (6)
O2—Mn—N1	95.16 (19)	O2—C6—C7	114.9 (6)
O3—Mn—N1	91.32 (19)	C5—C6—C7	121.5 (6)
O1—Mn—N1	91.7 (2)	C6—C7—H7A	109.5
O4—Mn—O5	88.09 (17)	C6—C7—H7B	109.5
O2—Mn—O5	85.76 (18)	H7A—C7—H7B	109.5
O3—Mn—O5	87.65 (18)	C6—C7—H7C	109.5
O1—Mn—O5	89.33 (18)	H7A—C7—H7C	109.5
N1—Mn—O5	178.6 (2)	H7B—C7—H7C	109.5
C4—O1—Mn	127.4 (4)	C9—C8—H8A	109.5
C6—O2—Mn	127.6 (4)	C9—C8—H8B	109.5
C9—O3—Mn	127.4 (4)	H8A—C8—H8B	109.5
C11—O4—Mn	127.2 (4)	C9—C8—H8C	109.5
C2—O5—Mn	128.0 (4)	H8A—C8—H8C	109.5
C2—O5—H5A	100 (6)	H8B—C8—H8C	109.5
Mn—O5—H5A	123 (6)	O3—C9—C10	122.8 (6)
N1—C1—S1	178.5 (7)	O3—C9—C8	114.3 (5)
O5—C2—H2A	109.5	C10—C9—C8	122.9 (6)
H5A—C2—H2A	136.0	C11—C10—C9	126.3 (6)
O5—C2—H2B	109.5	C11—C10—H10	116.8
H5A—C2—H2B	98.6	C9—C10—H10	116.8
H2A—C2—H2B	109.5	O4—C11—C10	124.2 (6)
O5—C2—H2C	109.5	O4—C11—C12	115.5 (6)
H5A—C2—H2C	91.3	C10—C11—C12	120.3 (6)
H2A—C2—H2C	109.5	C11—C12—H12A	109.5
H2B—C2—H2C	109.5	C11—C12—H12B	109.5
C4—C3—H3A	109.5	H12A—C12—H12B	109.5
C4—C3—H3B	109.5	C11—C12—H12C	109.5
H3A—C3—H3B	109.5	H12A—C12—H12C	109.5
C4—C3—H3C	109.5	H12B—C12—H12C	109.5
H3A—C3—H3C	109.5	C1—N1—Mn	162.9 (6)
H3B—C3—H3C	109.5		
			
O4—Mn—O1—C4	174.8 (5)	Mn—O1—C4—C5	9.1 (9)
O2—Mn—O1—C4	−11.4 (5)	Mn—O1—C4—C3	−173.5 (4)
N1—Mn—O1—C4	83.8 (5)	O1—C4—C5—C6	0.8 (11)
O5—Mn—O1—C4	−97.1 (5)	C3—C4—C5—C6	−176.4 (6)
O3—Mn—O2—C6	−175.2 (5)	Mn—O2—C6—C5	−1.7 (8)
O1—Mn—O2—C6	7.7 (5)	Mn—O2—C6—C7	−179.7 (4)
N1—Mn—O2—C6	−84.2 (5)	C4—C5—C6—O2	−4.7 (10)
O5—Mn—O2—C6	96.9 (5)	C4—C5—C6—C7	173.2 (6)
O4—Mn—O3—C9	0.7 (5)	Mn—O3—C9—C10	−1.7 (8)
O2—Mn—O3—C9	−173.2 (5)	Mn—O3—C9—C8	−179.9 (3)
N1—Mn—O3—C9	91.7 (5)	O3—C9—C10—C11	1.3 (10)
O5—Mn—O3—C9	−87.3 (5)	C8—C9—C10—C11	179.4 (6)
O3—Mn—O4—C11	0.8 (5)	Mn—O4—C11—C10	−1.3 (9)
O1—Mn—O4—C11	177.8 (5)	Mn—O4—C11—C12	177.7 (4)
N1—Mn—O4—C11	−90.5 (5)	C9—C10—C11—O4	0.3 (11)
O5—Mn—O4—C11	88.4 (5)	C9—C10—C11—C12	−178.7 (6)
O4—Mn—O5—C2	15.3 (5)	O4—Mn—N1—C1	5.2 (18)
O3—Mn—O5—C2	107.5 (5)	O3—Mn—N1—C1	−86.9 (18)
O1—Mn—O5—C2	−73.5 (5)	O1—Mn—N1—C1	94.0 (18)
O2—Mn—O5—C2	−164.7 (5)	O2—Mn—N1—C1	−174.6 (18)

### Hydrogen-bond geometry (Å, °)

**Table:**

*D*—H···*A*	*D*—H	H···*A*	*D*···*A*	*D*—H···*A*
O5—H5A···S1^i^	0.78 (7)	2.51 (6)	3.281 (4)	168 (7)

Symmetry codes: (i) *x*−1/2, −*y*+1/2, *z*−1/2.

## References

[bb1] Bruker (2000). *SADABS* Bruker AXS Inc., Madison, Wisconsin, USA.

[bb2] Bruker (2004). *APEX2* and *SAINT-Plus* Bruker AXS Inc., Madison, Wisconsin, USA.

[bb3] Sheldrick, G. M. (2008). *Acta Cryst.* A**64**, 112–122. 10.1107/S0108767307043930 18156677

[bb4] Stults, B. R., Day, R. O., Marianelli, R. S. & Day, V. W. (1975). *Inorg. Chem.* **14**, 722–730.

[bb5] Stults, B. R., Day, R. O., Marianelli, R. S. & Day, V. W. (1979). *Inorg. Chem.* **18**, 1847–1852.

[bb6] Swarnabala, G., Reddy, K. R., Tirunagar, J. & Rajasekharan, M. V. (1994). *Transition Met. Chem.* **19**, 506–508.

